# Resveratrol suppresses myofibroblast activity of human buccal mucosal fibroblasts through the epigenetic inhibition of ZEB1 expression

**DOI:** 10.18632/oncotarget.7763

**Published:** 2016-02-26

**Authors:** Yu-Chao Chang, Cheng-Wei Lin, Cheng-Chia Yu, Bing-Yen Wang, Yu-Hao Huang, Yang-Chih Hsieh, Yu-Liang Kuo, Wen-Wei Chang

**Affiliations:** ^1^ School of Dentistry, Chung Shan Medical University, Taichung, Taiwan; ^2^ Department of Dentistry, Chung Shan Medical University Hospital, Taichung, Taiwan; ^3^ School of Biomedical Sciences, College of Medical Science and Technology, Chung Shan Medical University, Taichung, Taiwan; ^4^ Institute of Oral Science, Chung Shan Medical University, Taichung, Taiwan; ^5^ Institute of Medicine, Chung Shan Medical University, Taichung, Taiwan; ^6^ Division of Throacic Surgery, Department of Surgery, Changhua Christian Hospital, Changhua County, Taiwan; ^7^ School of Medicine, National Yang-Ming University, Taipei City, Taiwan; ^8^ School of Medical Imaging and Radiological Sciences, Chung Shan Medical University, Taichung, Taiwan; ^9^ Department of Medical Imaging, Chung Shan Medical University Hospital, Taichung City, Taiwan; ^10^ Department of Medical Research, Chung Shan Medical University Hospital, Taichung City, Taiwan

**Keywords:** resveratrol, oral submucous fibrosis, ZEB1, EZH2, H3K27me3

## Abstract

Oral submucous fibrosis (OSF) is a precancerous condition of the oral mucosa without specific therapeutic drugs. We previously demonstrated that the zinc finger E-box binding homeobox 1 (ZEB1) plays a pathogenic role in the induction of the myofibroblast activity of buccal mucosal fibroblasts (BMFs) and contributes to the pathogenesis of OSF. Resveratrol is a natural polyphenolic flavonoid with anti-fibrosis activity in various tissues and has the capability to inhibit ZEB1 in oral cancer cells. We examined the effect of resveratrol on the myofibroblast activity of human primary fibrotic BMFs (fBMFs) derived from OSF tissues. With the collagen contraction assay, resveratrol displayed anti-myofibroblast activity in three fBMF lines. Resveratrol also inhibited the expression of fibrogenic genes at the mRNA and protein levels in a dose- and time-dependent manner. The downregulation of ZEB1 in fBMFs by resveratrol was mediated by epigenetic mechanisms, such as the upregulated expression of miR-200c and the enhancer of zeste homolog 2 (EZH2), as well as the trimethylated lysine 27 of histone H3 (H3K27me3). Resveratrol also increased the binding of H3K27me3 to the ZEB1 promoter. The knockdown of EZH2 in fBMFs caused the upregulation of ZEB1 and suppressed the inhibitory effect of resveratrol. Furthermore, the reversed expression pattern between EZH2 and ZEB1 was observed in 6/8 OSF tissues with twofold upregulation of ZEB1 expression compared with the adjacent normal mucosa. In conclusion, our data suggest that resveratrol epigenetically inhibits ZEB1 expression to suppress the myofibroblast activity of fBMFs and may serve as a dietary supplement for OSF patients.

## INTRODUCTION

Oral submucous fibrosis (OSF) is characterized as the submucosal accumulation of dense fibrous connective tissues with epithelial atrophy and infiltration of inflammatory cells [1]. According to epidemiological investigations, OSF is mainly caused by the habit of chewing areca quid [2]. According to a recent study in Taiwan, the incidence of malignant transformation among OSF patients was 3.7%, with an average duration of 37.42 months [3]. Meanwhile, the transformation rate was 7.6% after 17 years of follow-up in an Indian study [4]. These studies imply that OSF is a precancerous condition of the oral mucosa [1]. The pathogenesis of OSF is associated with the alteration of extracellular matrix components and chronic inflammation in the oral cavity [5, 6]. The activation of the tissue inhibitor of matrix proteinases [7] and plasminogen activator inhibitor-1 [8] has been demonstrated in OSF tissues and associated with decreased collagen degradation. The upregulation of proinflammatory cytokines, such as interleukin (IL)-6, IL-8, or tumor necrosis factor (TNF)-α, has been observed in OSF tissues [9] and linked to the profibrotic changes of the oral cavity. We have previously demonstrated that arecoline, the major alkaloid in areca quid [10], can induce myofibroblast transdifferentiation of buccal mucosal fibroblasts (BMFs) *via* the insulin-like growth factor-1 receptor of the zinc finger E-box binding homeobox 1 (ZEB1) signaling pathway [10]. The knockdown of ZEB1 by RNA interference inhibits the contraction of fibrotic BMFs (fBMFs) derived from OSF tissues [10]. With the observed upregulation of ZEB1 in OSF tissues [10], we hypothesize that the pharmaceutical inhibition of ZEB1 may benefit to OSF disease.

Resveratrol (3,5,40-trihydroxystilbene) is a natural polyphenolic flavonoid present in red grape, red wine, and other plant species with antioxidant, anti-inflammation, and anti-tumor activities [11]. Resveratrol has been shown to inhibit fibrosis of the lungs [12], liver [13, 14], or kidneys [15, 16]. We previously demonstrated that resveratrol could downregulate the expression of ZEB1 in head and neck squamous carcinoma cells [17]. In the present study, we demonstrated that resveratrol inhibited the myofibroblast phenotype and the expression of fibrotic genes of primary human fBMFs derived from OSF tissues. Resveratrol treatment of fBMFs induced the expression of miR-200c and the enhancer of zeste homolog 2 (EZH2) to trimethylate lysine 27 of histone 3 (H3K27me3). It also induced the binding of H3K27me3 on the ZEB1 promoter. The knockdown of EZH2 in fBMFs further increased the expression of ZEB1. Our data suggest that resveratrol can inhibit ZEB1 expression *via* epigenetic mechanisms and can be considered a potential therapeutic agent for OSF treatment.

## RESULTS

### Resveratrol inhibits the myofibroblast activity of fBMFs

**Figure 1:**
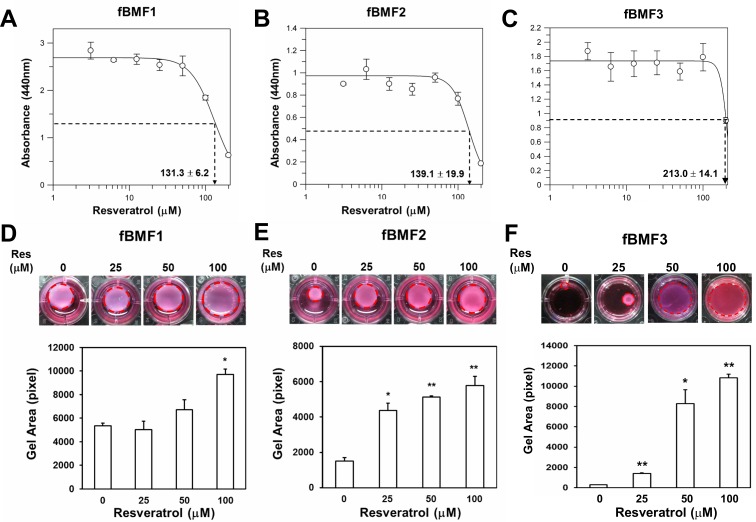
Resveratrol inhibits contraction activity of fibrotic BMFs **A.**, **B.**, **C.** Fibrotic BMF cell lines (fBMF1, fBMF2 or fBMF3) were seeded in wells of 96-well-plate as 1×10^4^ cells/well and treated with indicated concentration of resveratrol for 5 days (four replicates for each concentration). The cell survival/proliferation of fBMFs was determined by WST-1 reagent. IC_50_ values were calculated by GraFit software. **D.**, **E.**, **F.** The contraction activity of fBMF1, fBMF2 or fBMF3 was determined by collagen gel contraction assay (three replicates for each concentration). Images of gels were captured at Day 5 and gel areas (dotted circles) were calculated by ImageJ software. The experiments were repeated for three times and data from a representative experiment were presented. *, *p* < 0.05; **, *p* < 0.01 as comparison with non-resveratrol treated group.

Our group previously demonstrated that resveratrol, a natural polyphenolic flavonoid found in red wine [18], could suppress ZEB1 expression in oral squamous carcinoma cells [17]. In addition, resveratrol was demonstrated to reduce hepatic fibrosis in an experimental cirrhotic rat model [14]. Therefore, we hypothesized that resveratrol could also inhibit the myofibroblast activity of fBMFs. First, the effect of resveratrol on the cell proliferation of primary fBMFs was determined. After treatment with resveratrol for 5 days, the IC_50_ of resveratrol to three fBMF cell lines from different OSF patients (fBMF1, fBMF2, and fBMF3) was 131.3 ± 6.2, 139.1 ± 19.9, and 213.0 ± 14.1 μM, respectively (Figure 1A, 1B, and 1C). We examined if resveratrol could inhibit myofibroblast activity when the treatment concentration was below the IC_50_ value. In the collagen contraction assay, resveratrol decreased the gel volume of the three fBMFs in a dose-dependent manner and displayed a significant reduction of cell contraction capability in fBMF1 at 100 μM (Figure 1D), as well as in fBMF2 and fBMF3 at 25, 50, and 100 μM, respectively (Figure 1E and 1F).

### Resveratrol downregulates the expression of fibrogenic genes in fBMFs

**Figure 2:**
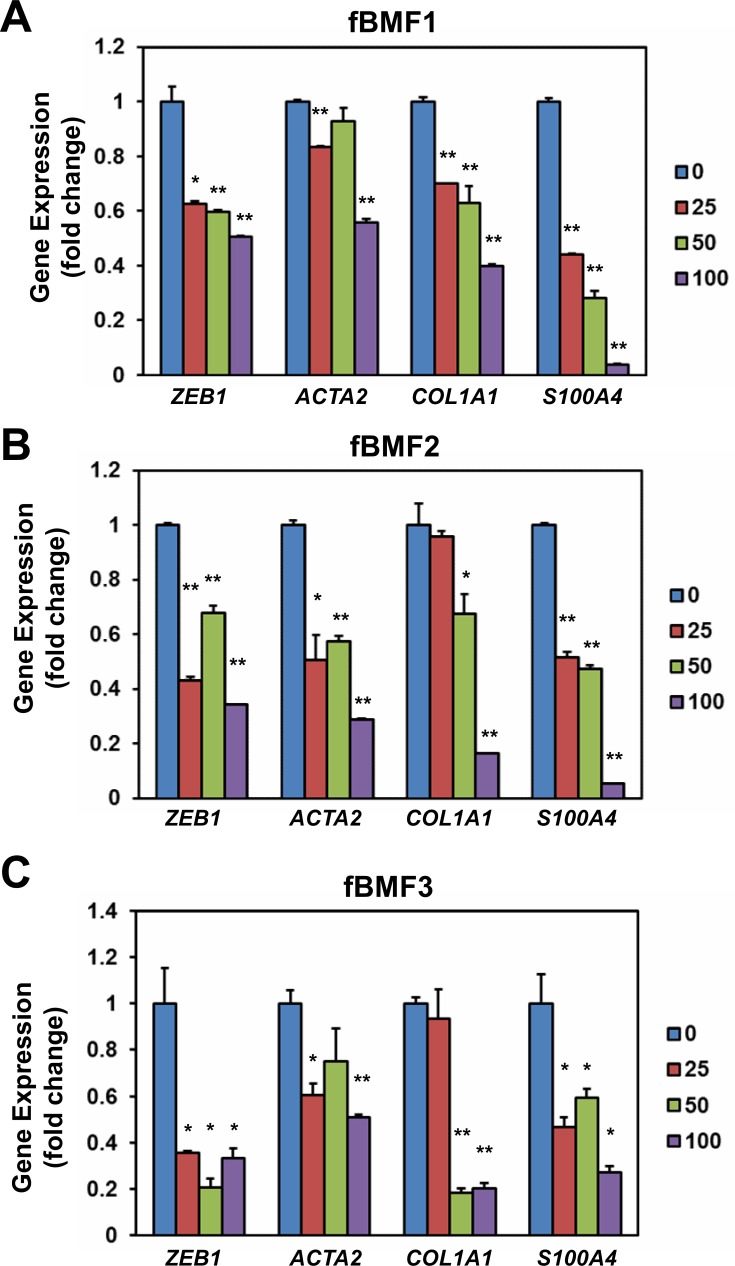
Resveratrol down-regulates the expression of fibrogenic genes of fibrotic BMFs 1×10^5^ cells/well of fibrotic BMF cell lines (fBMF1 **A.**, fBMF2 **B.** or fBMF3 **C.**) were seeded into 6-well-plate and cultured in 0.5 % FBS/DMEM at 37°C with 0, 25, 50, 100μM resveratrol for 48 hours (three replicates for each concentration). Total RNA were extracted and converted to cDNA. The expression of fibrogenic genes (*ZEB1*, *COL1A1*, *ACTA2*, *S100A4*) and an internal control gene (*MRPL19*) were determined by SYBR Green based quantitative PCR and presented as fold change to 0.1% ethanol treated group. The experiments were repeated for three times and data from a representative experiment were presented. *, *p* < 0.05; **, *p* < 0.01.

**Figure 3:**
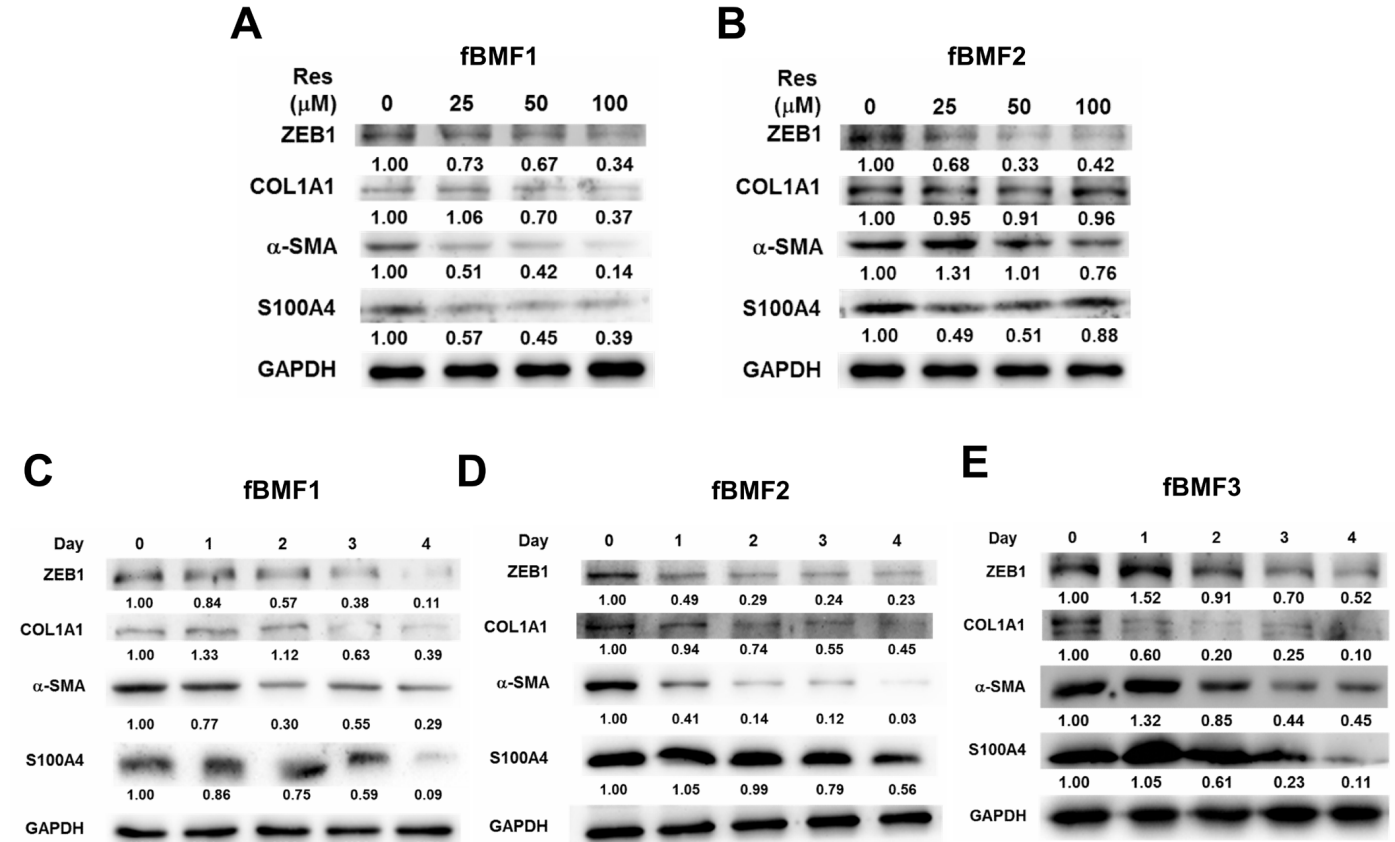
Resveratrol represses the expression of fibrogenic proteins of fibrotic BMFs 1×10^5^ cells/well of fibrotic BMF cell lines were seeded into 6-well-plate and cultured with 0.5% FBS/DMEM at 37°C with 0, 25, 50, 100 μM resveratrol for 48 hours **A.**, **B.** or with 100 μM resveratrol for 1 to 4 days **C.**, **D.**, **E.** Total proteins were extracted the expression of fibrogenic proteins were determined by western blot. The inserted numbers represented relative expression when compared with 0.1% ethanol treated group (A, B) or Day 0 group (C, D). The experiments were repeated for three times and data from a representative experiment were presented. *, *p* < 0.05; **, *p* < 0.01 as compared to the data from 0.1% ethanol treated group.

We examined the effect of resveratrol on the expression of fibrogenic genes in fBMFs. As shown in Figure 2, resveratrol downregulated the mRNA expression of *ZEB1*, *ACTA2*, *COL1A1*, and *S100A4* in three fBMFs at 48 h (Figure 2) in a dose-dependent manner. The protein expression of ZEB1, -SMA, or S100A4 was also downregulated in fBMF1 and fBMf2 at 48 h post resveratrol treatment in a dose-dependent manner (Figure 3A and 3B). COL1A1 protein was downregulated in fBMF1 (Figure 3A). We further examined the effect of 100 μM resveratrol in the expression of fibrogenic proteins in a time course treatment. The protein expression of ZEB1, COL1A1, -SMA or S100A4 was time-dependently inhibited by resveratrol in three fBMF cell lines (Figure 3C to 3E). From these results, we discovered that resveratrol displayed an inhibitory effect in myofibroblast activity and fibrogenic gene expression in OSF tissue-derived fBMFs.

### Effects of resveratrol in DNA methylation status of ZEB1 promoter

**Figure 4:**
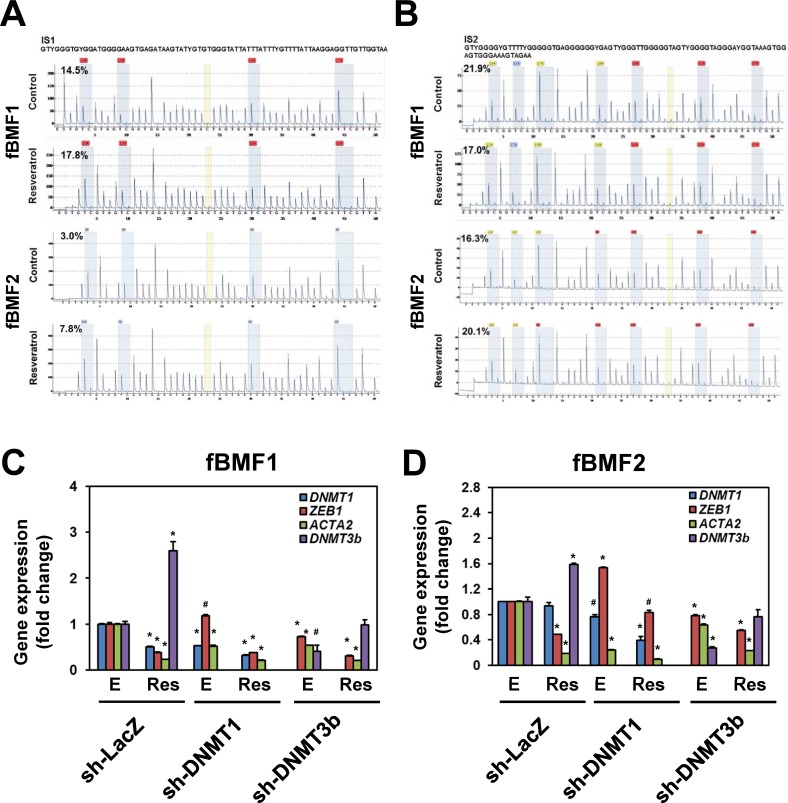
The effects of DNA methylation in resveratrol mediated ZEB1 down-regulation in fibrotic BMFs **A.**, **B.** 1×10^6^ cells/dish of fibrotic BMF cell lines were seeded into 10 cm dishes and cultured at 37°C with 100 μM resveratrol for 24 hours. The genomic DNA were extracted and 1 μg was taken for bisulfite reaction. DNA methylation was determined by pyrosequencing and presented as percentage of methylated CpG at each site. The average percentage of methylated CpG of island 1 (IS1) or island 2 (IS2) of control or resveratrol treated group was indicated. **C.**, **D.** fBMF1 or fBMF2 were transduced with sh-LacZ, sh-DNMT1 or sh-DNMT3b lentivirus and selected with 2 μg/ml puromycin for three days followed by treatment of 0.1% enthanol **E.** or 100 μM resveratrol (Res) for 48 hours. The expression of *DNMT1*, *ZEB1*, *ACTA2* or *DNMT3b* was determined by quantitative RT-PCR. The experiments were repeated for two times and data from a representative experiment were presented. #, *p* < 0.05; *, *p* < 0.01 as compared between E and Res group of each shRNA-lentivirus transduced samples.

We investigated how resveratrol downregulated ZEB1 expression in fBMFs and firstly focused on its promoter methylation status. Through bisulfite pyrosequencing, we determined that resveratrol treatment only slightly increased DNA methylation in the sequenced CpG island fragment 1 (IS1) within the ZEB1 promoter region of both fBMF1 and fBMF2 (Figure 4A; the average CpG methylation ratio in the ethanol control *versus* the resveratrol-treated group changed from 14.5% to 17.8% in fBMF1 or 3.0% to 7.8% in fBMF2). Similarly, resveratrol only slightly increased in the CpG island fragment 2 (IS2) in fBMF2 (Figure 4B, lower panel; average CpG methylation ratio in ethanol control *versus* resveratrol-treated group changed from 16.3% to 20.1%) but it was decreased in fBMF1 (Figure 4B, upper panel; average CpG methylation ratio in ethanol control *versus* resveratrol-treated group changed from 21.9% to 17.0%). We used shRNA-mediated gene silencing to knockdown DNA methyltransferase DNMT1 or DNMT3b to further determine the role of DNA methylation in the resveratrol-mediated ZEB1 downregulation. As shown in Figure 4C and 4D, the knockdown of DNMT1 or DNMT3b in fBMF1 (Figure 4C) or fBMF2 (Figure 4D) did not influence the inhibitory effect of resveratrol on ZEB1 transcription. From these results, the downregulation of ZEB1 may not be mainly caused by the increased DNA methylation of the ZEB1 promoter.

### Resveratrol induces miR-200c expression in fBMFs

**Figure 5:**
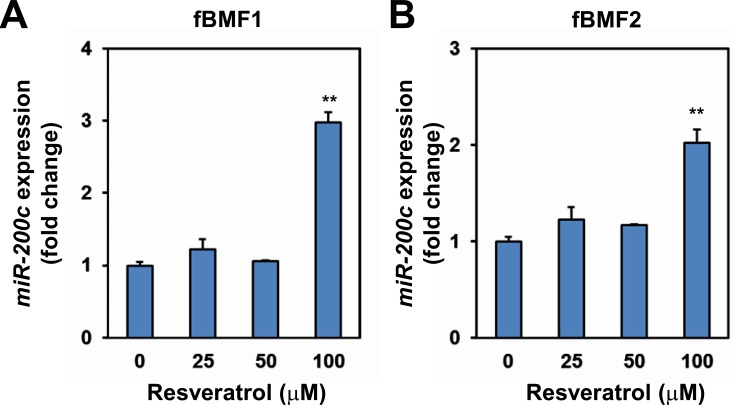
The effect of resveratrol in miR-200c expression of fibrotic BMFs fBMF1 **A.** or fBMF2 **B.** cells were treated with 0, 25, 50, 100 μM resveratrol for 48 hours and total RNA were extracted. miR-200c expression was determined by quantitative RT-PCR method and presented as fold change of ethanol treated group. The experiments were repeated for two times and data from a representative experiment were presented.**, *p* < 0.01 as compared to non-resveratrol treated group.

ZEB1 is a well-known target of miR-200c [19]. Thus, we examined if resveratrol upregulated miR-200c expression in fBMFs. Quantitative RT-PCR (qRT-PCR) analysis indicated that resveratrol treatment significantly increased miR-200c expression in two fBMF cell lines compared with the ethanol control (Figure 5A and 5B).

### Resveratrol induces EZH2 and H3K27me3 expression to inhibit ZEB1 transcription

**Figure 6:**
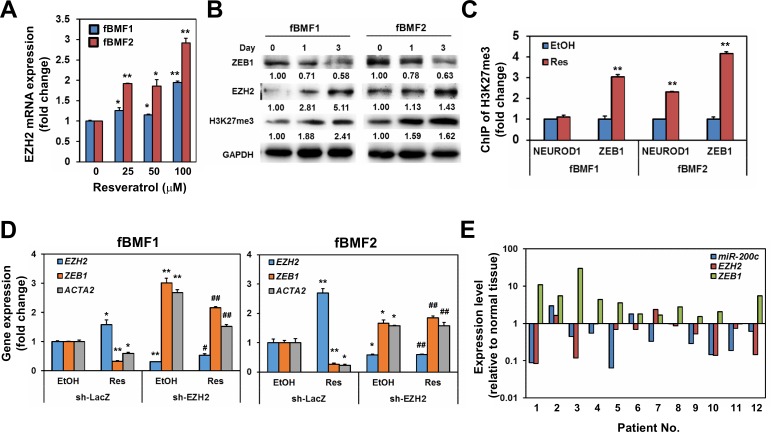
EZH2 is the key molecule in resveratrol mediated suppression of ZEB1 expression in fibrotic BMFs **A.** The mRNA expression of EZH2 in resveratrol treated fBMF1 or fBMF2 were determined by SYBR-Green based quantitative RT-PCR. *, *p* < 0.05; **, *p* < 0.01 as compared to the data from non-treated group. **B.** Protein expression of ZEB1, EZH2 or H3K27me3 in resveratrol treated fBMF1 or fBMF2 was determined with western blot. Inserted numbers indicated relative expression level in comparison with Day 0. **C.** fBMF1 or fBMF2 were serum-starved for 24 hours, treated with 0.1% ethanol (EtOH) or 100 μM resveratrol (Res). The binding of H3K27me3 on neuronal differentiation 1 (NEUROD1) or ZEB1 promoter was determined by chromatin immunoprecipitation and quantitative PCR. Data were presented as fold change of EtOH treated group. **, *p* < 0.01 as compared to the data from non-treated group. **D.** The mRNA expression of *EZH2* or *ZEB1* in fBMF1 or fBMF2 after lentivirus-mediated EZH2 knockdown was determined by SYBR-Green based quantitative RT-PCR. *, *p* < 0.05; **, *p* < 0.01 as compared to the data from EtOH treated sh-LacZ transduced cells. #, *p* < 0.05; ##, *p* < 0.01 as comparison with the data from Res treated sh-LacZ transduced cells. The experiments (A-D) were repeated at least two times and data from a representative experiment were presented. **E.** The expression of *EZH2*, *ZEB1* and *miR-200c* in OSF tissues and their adjacent normal mucosa was determined by qRT-PCR. Data were presented as fold change in comparison to adjacent normal mucosa.

**Figure 7:**
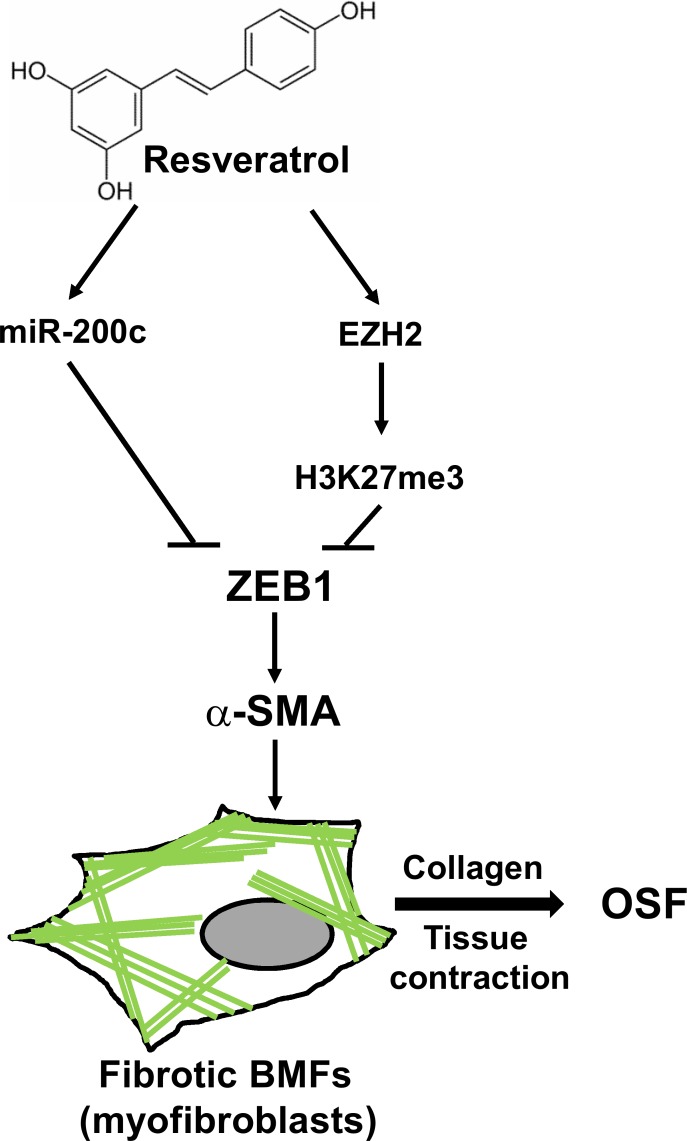
The proposed model of the putative therapeutic effect of resveratrol in OSF disease The expression of ZEB1 in BMFs could induce the formation of stress fibers which comprised of α-SMA (green lines) and lead to the transdifferentiation into myofibroblasts to secret collagen and cause tissue contraction of oral cavity. Treatment of resveratrol could upregulate the expression of miR-200c and EZH2/H3K27me3 to repress ZEB1 expression and leads to the inhibition of myofibroblast properties in fibrotic BMFs and may benefit to OSF disease, the pre-cancerous lesion of oral cavity.

In addition to miR-200c, H3K27me3 is associated with the downregulation of ZEB1 expression [20]. In a study on dermal fibroblasts, the inhibition of H3K27me3 could promote dermal fibrosis [21]. EZH2 is the histone methyltransferase for the trimethylation of H3K27; thus, we examined if resveratrol upregulated the expression of EZH2 in fBMFs. With qRT-PCR analysis, resveratrol treatment increased the expression of EZH2 in fBMF1 and fBMF2 in a dose-dependent manner (Figure 6A). The protein expression of EZH2 and H3K27me3 were also upregulated, whereas ZEB1 was repressed by resveratrol in both fBMF1 and fBMF2 in a time-dependent manner (Figure 6B). The binding of H3K27me3 plays a transcriptional repression role to its genes [22], we next using chromatin immunoprecipitation, a method being widely used to examine the interactions between a specific protein and a genomic DNA region [23], to examine the binding of H3K27me3 in ZEB1 promoter region of resveratrol-treated fBMFs. After extraction of immunoprecipiated DNA with H3K27me3 antibody and detection the H3K27me3 DNA binding sequence within ZEB1 promoter with quantitative PCR, results revealed that the treatment of resveratrol significantly increased the binding of H3K27me3 to the ZEB1 promoter region in both fBMF1 and fBMF2 (Figure 6C). We further examined if the suppression of EZH2 expression in fBMFs would affect the expression of ZEB1 and influence the inhibitory effect of resveratrol on ZEB1 expression. The lentivirus-mediated EZH2-specific shRNA delivery led to the knockdown of EZH2 and significantly increased the mRNA expression of ZEB1 in fBMF1 and fBMF2 cells without resveratrol treatment (Figure 6D). The inhibitory effect of resveratrol on ZEB1 expression in fBMF1 and fBMF2 was also suppressed by the knockdown of EZH2 (Figure 6D), as well as the treatment of 3-Deazaneplanocin A (DZNep), an inhibitor of EZH2 (Figure S1). Finally, we determined the expression of ZEB1 and miR-200c in 12 OSF tissues and their adjacent normal oral mucosa. A total of 8/12 (66.7%) specimens displayed the upregulation of ZEB1 as a fold increase larger than 2 in OSF tissues compared with the adjacent normal mucosa (Figure 6E). Among the 8 cases, 6 specimens displayed downregulation of either EZH2 or miR-200c (Figure 6E). From these results, we hypothesized that resveratrol could inhibit the myofibroblast properties of fBMFs derived from OSF tissues, including the contraction activity and expression of fibrotic genes (α-SMA, collagen, or S100A4) through the epigenetic repression of ZEB1, which was composed of the upregulated miR-200c and EZH2/H3K27me3 expression (Figure 7).

## DISCUSSION

In this study, we determined that resveratrol inhibited the fibrotic features of fBMFs derived from OSF tissues *via* the epigenetic inhibition of ZEB1: the upregulation of miR-200c and EZH2/H3K27me3 expression. The miR-200 family, which consists of miR-200a, miR-200b, miR-200c, miR-141, and miR-429, is known for its capability to regulate epithelial-mesenchymal transition (EMT) program in cancer cells *via* the posttranscriptional repression of ZEB1 or ZEB2 [24]. In addition to ZEB1, resveratrol inhibited ZEB2 mRNA expression at a concentration of 100 μM in fBMF1 or fBMF2 cells (Figure S2). Therefore, ZEB2 may also be involved; however, this hypothesis requires further investigation. The EMT change of renal tubular epithelial cells is considered one of the mechanisms of renal fibrosis [25]. The tumor growth factor (TGF)-β1 was used to induce EMT of renal tubular epithelial cells, and the downregulation of the miR-200 family was observed *via* a Smad signaling-dependent mechanism [26]. The repression of the miR-200 family could also cause EMT of the renal tubular epithelium, which could be reversed by the knockdown of ZEB1 [26]. The downregulation of the miR-200 family by TGF-β1 could also be observed in human biliary epithelial cells as a model of liver fibrosis [27]. In our study, we observed that resveratrol inhibited the fibrotic features of fBMFs and could upregulate the expression of miR-200c (Figure 4B and 4C). In OSF tissues, the expression levels of ZEB1 and miR-200c also displayed a reverse correlation (Figure 6E). These results suggest that the miR-200 family may protect against OSF disease. The expression of miRNAs could be regulated by nuclear receptor signaling, such as peroxisome proliferator-activated receptors (PPARs) [28]. Recently, Zhou et al. demonstrated that resveratrol could up-regulate PPARα expression to prevent renal lipotoxicity in an obesity mouse model [29]. The role of PPARα in the up-regulation of miR-200c in resveratrol treated fBMFs remains to be further investigated. We previously demonstrated that S100A4 expression was up-regulated in OSF tissues and knockdown of S100A4 could inhibit arecoline-induced myofibroblast transdifferentiation in BMFs [30]. Here we observed that resveratrol also inhibited S100A4 expression (Figure 2 and Figure 3). By bioinformatics analysis, there are several putative E-box domains, the ZEB1 binding motif, within S100A4 promoter (Figure S3). It indicates that S100A4 may regulate by ZEB1 but remains to be further investigated.

EZH2 belongs to the polycomb group of proteins, which function as transcriptional repressors through the formation of polycomb-repressive complexes [31]; this histone methyltransferase catalyzes H3K27me3 [32]. Chaffer et al. [20] demonstrated that the ZEB1 promoter could be converted into the active chromatin configuration in breast cancer cells by removing the binding of H3K27me3 in response of TGF-β1 treatment. The treatment of dermal fibroblasts with DZNep, an inhibitor of EZH2, caused the inhibition of H3K27me3 and the induction of the profibrotic transcription factor fra-2, which led to dermal fibrosis [21]. We determined that resveratrol could induce the expression of EZH2 and H3K27me3 to repress ZEB1 transcription. We observed a reverse correlation between ZEB1 and EZH2 expression (Figure 6E). Therefore, OSF may be associated with the downregulation of EZH2 and H3K27me3.

Several studies have reported the benefits of resveratrol in various diseases, including heart diseases, aging-associated diseases, neurodegeneration, and cancers [33]. Resveratrol is known to activate the anti-aging deacetylase enzyme sirtuin 1 [34]. The activation of sirtuin 1 by resveratrol has been demonstrated to ameliorate tissue fibrosis in dystrophic cardiomyopathy [35] or systemic sclerosis [36]. We provide another novel mechanism of the anti-fibrotic effect of resveratrol *via* the epigenetic inhibition of ZEB1. Another benefit of resveratrol in OSF disease is its anti-inflammatory activity because of the altered cytokine profiles in OSF tissues [5, 9]. Resveratrol has been demonstrated to reduce the production of pro-inflammatory cytokines, such as IL-6 and TNF-α, in severe burns [37], hepatic inflammation [38], or trauma-hemorrhage-induced injury [39]. Meanwhile, the reduced level of anti-oxidants, such as beta carotene and vitamin E, and an increased level of lipid peroxidation products in plasma were observed in OSF patients, which suggested the pathological role of oxidative stress in OSF disease [40]. The antioxidant activity of resveratrol has been studied extensively [33, 41]. The pathologic factors of OSF disease include the increased level of pro-inflammatory cytokines in OSF lesions [9] and oxidative stress; thus, the multiple anti-inflammation and antioxidant activities of resveratrol and the reduction of ZEB1 expression allow this compound to serve as a potential dietary supplement for OSF patients to benefit their fibrotic condition and prevent the occurrence of oral squamous carcinomas. One of the limitations of our study is the high concentration used (25 μM to 100 μM), which makes entering into clinical development difficult. A number of resveratrol analogs are being developed to improve the *in vivo* efficiency of resveratrol *via* the structural analysis of resveratrol and its targets [42, 43]. The efficiency of resveratrol analogs in suppressing the myofibroblast activity of fBMFs derived from OSF tissues is worthy of further examination.

## MATERIALS AND METHODS

### Isolation of BMFs and cell culture

Fibrotic BMFs (fBMFs) were isolated from OSF tissues of three OSF patients with an areca quid chewing habit in the Oral Medicine Center (Chung Shan Medical University Hospital, Taichung, Taiwan). The patients were enrolled with informed consent and followed the protocol approved by Institutional Review Board of Chung Shan Medical University Hospital. Surgical OSF tissues were sliced into 1mm^3^ pieces and digested with collagenase/hyaluronidase solution (STEMCELL Technologies Inc., Vancouver, Canada) at 37°C for 2 hours. The released BMFs were then collected and washed with DMEM, were maintained in DMEM medium containing 10% fetal bovine serum at 37°C incubator with 5% CO_2_ and were characterized according to the morphology with spindle shape. For determination of the cytotoxic effect of resveratrol in fBMFs, cells were seeded as 1 × 10^4^/100μl/well in 96-well-plate in presence of different concentration of resveratrol or 0.1% ethanol. Cell proliferation was determined by WST-1 reagent (Roche Life Science, Indianapolis, IN, USA) at Day 5 post treatment and IC_50_ values were calculated by GraFit software (Erithacus Software Ltd., West Sussex, UK).

### Reagents and antibodies

Resveratrol was purchased from Sigma-Aldrich (St. Louis, MO, USA) and was dissolved in ethanol as 100 mM stock. Monoclonal mouse anti-human antibody against α-SMA (1A4) and rabbit polyclonal anti-human antibody against COL1A1 (H-197) were purchased from Santa Cruz Biotechnology, Inc. (Santa Cruz, CA, USA). Rabbit polyclonal anti-human antibodies against ZEB1, S100A4 and GAPDH and horseradish peroxidase-conjugated anti-mouse IgG or anti-rabbit IgG antibodies were purchased from GeneTex, Inc. (Hsinchu City, Taiwan). Mouse monoclonal anti-human EZH2 antibody was purchased from BD Transduction Laboratories (San Jose, CA, USA). Rabbit polyclonal anti-human H3K27me3 antibody was purchased from Merck Millipore (Darmstadt, Germany). A collagen solution from bovine skin was purchased from Sigma-Aldrich.

### Collagen contraction assay

fBMFs (2×10^5^) were suspended in 0.5 ml of a 2 mg/ml collagen solution (Sigma-Aldrich) and added into one well of a 24-well plate. The plate was incubated at 37°C for 2 hours, which caused polymerization of the collagen gels. After detaching gels from the wells, the gels were further incubated in 0.5 ml MEMα medium with or without resveratrol treatment for 5 days. The contraction of the gels was evaluated by photographing the gels and using ImageJ software (National Institutes of Health, Bethesda, MD, USA) to calculate the gel area after contraction.

### Quantitative real-time RT-PCR

Total RNA was extracted using a Quick RNA MiniPrep kit (Zymo Research, Irvine, CA) and reverse transcribed to cDNA using oligo(dT) primer (RevertAid First Strand cDNA Synthesis Kit, Thermo Fisher Scientific Inc., Waltham, MA, USA). RT-PCR for simultaneous detection and quantification of the cDNA samples was performed on an ABI StepOnePlus™ Real-Time PCR System and analyzed with the StepOne software (Applied Biosystems, Life Technologies Corp., Carlsbad, CA, USA). Fifty nanograms of cDNA sample were used in a SYBR Green-based qPCR reaction (Kapa Biosystems, Inc., Wilmington, MA, USA) ; the cycling conditions were as follows: 50°C for 2 min, 95°C for 10 min, followed by 40 cycles of 95°C for 10 sec and 60°C for 1 min. The end-point used in the real-time quantification was calculated by the StepOne software, and the threshold cycle number (Ct value) for each analyzed sample was calculated. Each target gene was normalized to MRPL19, which was reported as one of the most stable internal control gene among literatures [44-46], to derive the change in Ct value (ΔCt). The relative gene expression differences between groups were calculated by 2^−ΔΔCt^ [47]. Primer sequences used in this study were listed as follow:

*COL1A1*: 5′-GGGTGACCGTGGTGAGA-3′ and 5′-CCAGGAGAGCCAGAGGTCC-3′;

*ACTA2*: 5′-AGCACATGGAAAAGATCTGGCACC-3′ and 5-TTTTCTCCCGGTTGGCCTTG-3′;

*ZEB1*: 5′-AGCAGTGAAAGAGAAGGGAATGC-3′ and 5′-GGTCCTCTTCAGGTGCCTCAG-3′;

*S100A4*: 5′-GAGCTGCCCAGCTTCTTG-3′ and 5′-TGCAGGACAGGAAGACACAG-3′;

*EZH2*: 5′-GACTGGCGAAGAGCTGTTTT-3′ and 5-TCTTTCGATGCCGACATACTT-3′;

*DNMT1*: 5′-CCCCTGAGCCCTACCGAAT-3′ and 5′-CTCGCTGGAGTGGACTTGTG-3′;

*DNMT3b*: 5′-TTGAATATGAAGCCCCCAAG-3′ and 5′-TGATATTCCCCTCGTGCTTC-3′;

*MRPL19*: 5′-GGGATTTGCATTCAGAGATCAG-3′ and 5′-GGAAGGGCATCTCGTAAG-3′

Bulge-Loop^TM^ miRNA qRT-PCR primer set (RiboBio Co., Ltd., Guangzhou, China) was used for detection of miR-200c expression. Briefly, 0.2 μg total RNA was used for reverse transcription by miR-200c specific RT primer at 42°C for 60 mins. miR-200c expression was determined by SYBR Green reagent and specific Bulge-Loop^TM^ forward and reverse primers by following cycling reaction: 95°C for 20 sec followed by 40 cycles of 95°C for 10 sec, 60C for 20 sec and 70C for 10 sec. miR-200c expression of each group was normalized to U6 snRNA and calculated the fold changes in comparison with 0.1% EtOH treated samples.

### Western blot analysis

Cells were lysed with NP-40 lysis buffer, and protein concentration was determined by BCA protein assay reagent (Thermo Fisher Scientific Inc., Rockford, IL). A total of 25 μg of total protein were separated by SDS-PAGE and transferred to a PVDF membrane (Millipore, Billerca, MA). Protein detection was conducted by the SignalBoost^TM^ Immunodetection Enhancer kit (Calbiochem, San Diego, CA) according to the manufacturer's recommendation. Briefly, the primary antibody was diluted with a primary antibody solution and incubated with the PVDF membrane at 4°C overnight. After washing with 0.1% Tween-20 in a Tris buffer solution, the membrane was incubated for 1 hour at room temperature with a secondary antibody that was diluted with secondary antibody buffer. The signals were developed using an ECL-plus chemiluminescence substrate (Perkin-Elmer, Waltham, MA) and captured using a LAS-1000plus Luminescent Image Analyzer (GE Healthcare Biosciences, Piscataway, NJ). The band intensity was quantified using Bio1D software (Vilber Lourmat, Marne-la-Vallée, France).

### Bisulfite pyrosequencing analysis of DNA methylation

Genomic DNA was extracted by Quick-gDNA™ MiniPrep kit (Zymo Research) and sonicated into 1 kb fragments. Sonicated genomic DNA fragments were treated with NaOH for 15 mins and performed bisulfite conversion by adding hydroquinone and sodium metabisulfite at 56°C for 16 hours. After bisulfite reaction, the genomic DNA were further purified by Quick-gDNA™ MiniPrep kit, treated with NaOH, precipitated by ethanol and dissolved in TE buffer. The prediction of CpG island within ZEB1 promoter region and the sequencing primers using in pyrosequencing were designed by PyroMark Q24 Advanced Software 3.0 (Qiagen GmbH, Hilden, Germany). Pyrosequencing was performed by Genomics BioSci & Tech. Ltd. (New Taipei City, Taiwan).

### Chromatin immunoprecipitation

Cells were harvested by trypsin-EDTA and fixed with 1% formaldehyde at room temperature for 10 min. After quenching the formaldehyde with 125 mM glycine, the cells were lysed with mammalian cell lysis buffer (Pierce, Thermo Fisher Scientific, Inc.) and treated with micrococcal nuclease (Fermentas, Thermo Fisher Scientific, Inc.) at 37°C for 20 min. The fragmented DNA solution was further diluted with ChIP dilution buffer (0.01% SDS, 1.1% Triton X-100, 1.2 mM EDTA, 16.7 mM Tris-HCl and 167 mM NaCl) and pre-cleared by incubation with 10 μl Protein A Mag Sepharose (GE Healthcare) at room temperature for 2 hours. After the pre-clear step, 1 g of anti-H3K27me3 antibody or normal rabbit IgG was added to the cell lysate, and the mixture was incubated at 4°C overnight. After washing, protein/DNA complexes were eluted using elution buffer (1% SDS and 100 mM NaHCO_3_). Reverse crosslinking of the protein/DNA complexes was performed by treating with 200 mM NaCl at 65°C for at least 5 hours, and the proteins were digested with proteinase K. The DNA was further purified using a Wizard^TM^ PCR Clean-Up kit (Promega, Madison, WI, USA). The H3K27me3 binding region in the promoter of ZEB1 or NEUROD1 was further detected using SYBR Green quantitative PCR method by the primer sets described by Chaffer CL et al. [20].

### Lentivirus-based shRNA delivery

The lentiviral vectors carrying LacZ-specific shRNA (sh-LacZ, TRCN0000231722; sh-DNMT1, TRCN0000021890, TRCN0000021891; sh-DNMT3b, TRCN0000035684, TRCN0000035685; EZH2 specific shRNA (sh-EZH2, TRCN0000040073, TRCN0000040076 and TRCN0000010475) were obtained from the National RNAi Core Facility at the Institute of Molecular Biology (Academia Sinica, Taipei, Taiwan), and shRNA lentiviruses were produced in 293T cells by co-transfection with package plasmids (pMD.G and pCMVDR8.91). For DNMT1, DNMT3b or EZH2 knockdown experiments, two (DNMT1 or DNMT3b) or three (EZH2) gene specific shRNA virus were mixed for transduction into fBMFs. For virus transduction, cells were plated at 2×10^5^ cells per well in six-well plates and transiently transduced with lentivirus (MOI = 1) in the presence of 8 μg/ml polybrene (Sigma-Aldrich) for 24 hours. The cells then were selected with 2 μg/ml puromycin (Sigma-Aldrich) and were harvested at 96 hours post-transduction for subsequent analyses.

### Statistical analysis

Quantitative data were presented as the mean ± SD, and the comparisons between groups were analyzed with a two-tailed, nonparametric Student's *t*-test. A p value of less that 0.05 was considered significantly different.

## SUPPLEMENTARY INFORMATION FIGURES



## Abbreviations

fBMFs: fibrotic buccal mucosal fibroblasts;
OSF: oral submucous fibrosis;
ZEB1: zinc finger E-box binding homeobox 1;
EZH2: enhancer of zeste homolog 2;
H3K27me3: trimethylated lysine 27 of histone H3.
