# Supplementary material for: Resveratrol suppresses myofibroblast activity of human buccal mucosal fibroblasts through the epigenetic inhibition of ZEB1 expression
Source: Oncotarget. 2016 Feb 26;7(11):12137–49. doi: 10.18632/oncotarget.7763 (PMC4914274; doi:10.18632/oncotarget.7763)
Supplement: Supplementary file 1 [file oncotarget-07-12137-s001.pdf]

Resveratrol suppresses myofibroblast activity of human buccal mucosal fibroblasts through the epigenetic inhibition of ZEB1 expression

Supplementary Information

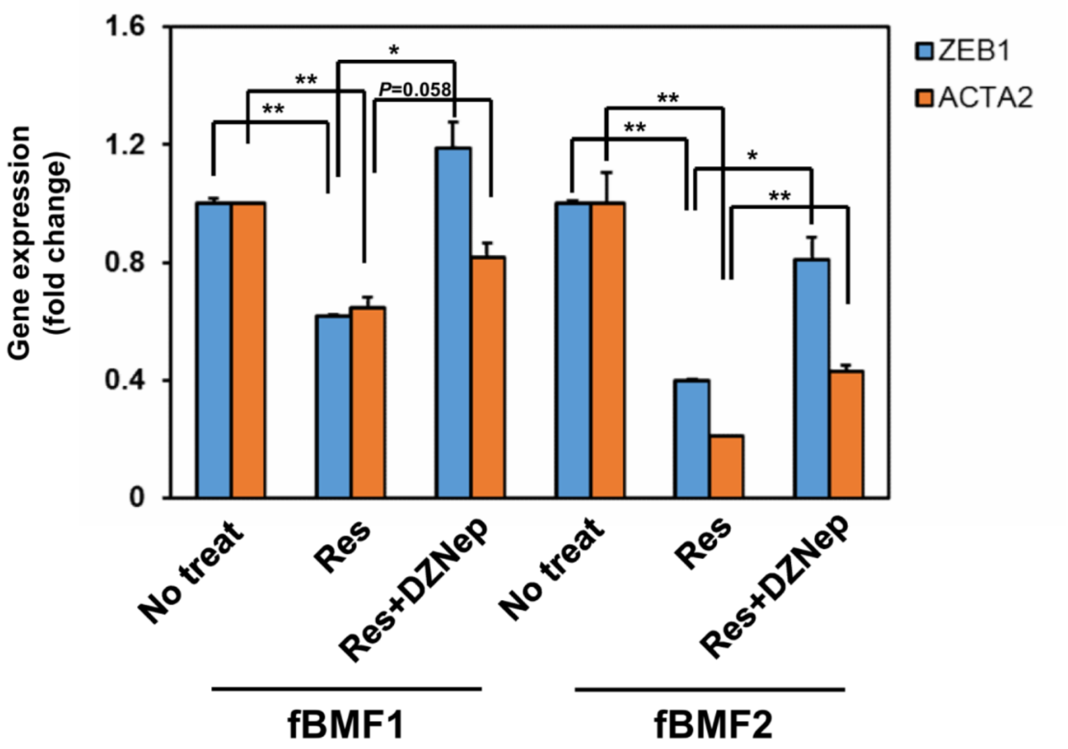

Figure S1: 3-Deazaneplanocin A (DZNep) reverses the inhibitory effect of resveratrol on ZEB1 expression. fBMF1 or fBMF2 cells were seeded into 6-well-plate as 1×10^5 cells/well and cultured in 0.5 % FBS/DMEM at 37°C with 0.1% ethanol/DMAO (No treat), 0.1%DMSO/100 μM resveratrol (Res), or 100μM resveratrol/10μM DZNep for 48 hours. The expression of *ZEB1* and *ACTA2* was determined by SYBR-Green based qRT-PCR method. *, $p < 0.05$; **, $p < 0.01$.

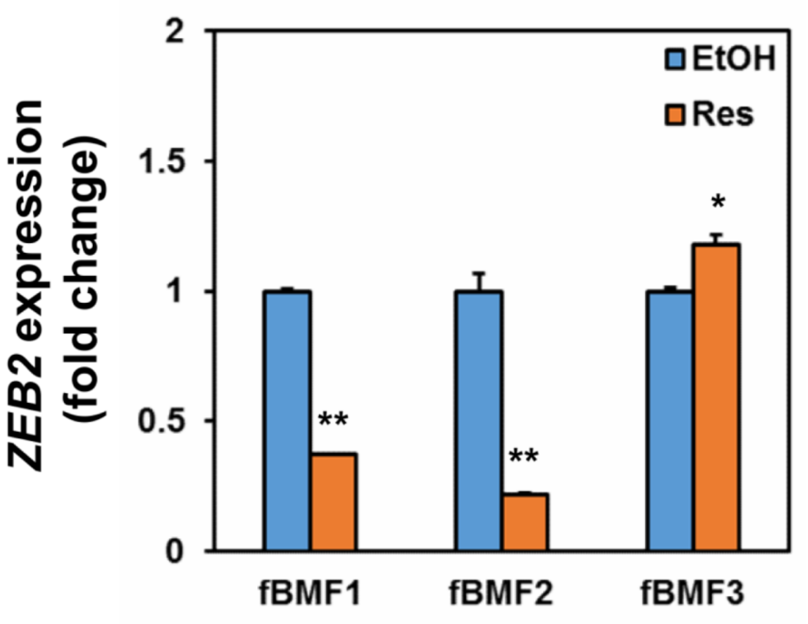

Figure S2: ZEB2 expression in resveratrol treated fibrotic BMFs. fBMF1, fBMF2 or fBMF3 cells were seeded into 6-well-plate as 1×10^5 cells/well and cultured in 0.5 % FBS/DMEM at 37°C with 0.1% ethanol (EtOH) or 100 μM resveratrol (Res) for 48 hours. The expression of *ZEB2* was determined by SYBR-Green based qRT-PCR method. *, $p< 0.05$; **, $p< 0.01$ as comparison between EtOH and Res.

IUPAC/Regular Expression Analysis Results

Results for your IUPAC string (*CANNTG*) search (match highlighted):

```
>S100A4:chr1:150345377 [-700..299](-) [human, Homo sapiens]

ATGGGGATCCCCACCCCAGTTTTTGTTTCTGAATCTTTATTTTTTTAAGA     50
GACAAGGTCCTCTGTGTTGCTCAGGCTGGAGAGCAGTGGCTTGAGCATAG    100
CCAACTGCAGTCTCGAACTCCTGGGCTCAAATGATCCTCCTGTCTCAGCT    150
TCCTGACTAGCTGGGACTACAGGCTACAGCCATGCTGCCCAGCTAATTAA    200
AAAAAAAAATTGTTTTTCCTTTTTATAGAGACAGAAGTCTCTCTATGTTG    250
CCTAGGCTGGTCTTGAACTCCTGGCCTCAGGCGATCCTCCCATCTCCCCC    300
CTAGCTTTTGTGTCACCACATTTCCAGGGCAATCTCCCACCTGTCACCCA    350
CCACCCCCTGCATCTCCTTTCCTAGGTCCCCATGGGACTACTCCCTGTCC    400
CCCATGCTCCAGGCACAGGCTGCCCCTTCCTCCACCTCTCTAAAACTCAG    450
GCTGAGCTATGTACACTGGGTGGTGCCCATCTCATCCAGTCCCCTGCTAG    500
TAACCGCTAGGGCTTACCCGTTACCCACGGGTGCCCACCTGGGAACAGGA    550
GGCTTGGTTCCACGGCTGGGCTGGTGGAGGGTGCTGTGGCACTTACCGCA    600
TCAGCCCACAGCAGGAAGGCAGTATCCGCTCTCCCCTGTCCCCTGCTATG    650
GGCAGGGCCTGGCTGGGGTATAAATAGGTCAGACCTCTGGGCCGTCCCCA    700
TTCTTCCCCTCTCTACAACCCTCTCTCCTCAGCGCTTCTTCTTTCTTGGT    750
TTGGTGAGTTGTGTTGGCCTGACTGGCATGCAAGGGGTGTCAGAGGCCAG    800
GGCTGGGGAAGGAGAAGGGGAGGCTGGTGGGGGCCAGATGTGCTAAAGAG    850
ATCCAGATGTGAGATTCTGATGTGGAACTCTGGGTGGATTGTGTGCGTGG    900
GTGTGCATGGCACACACACACATGCACGTAAGACGGAGGAAAAAACAAAC    950
AGAAAAGTGAGCAAGTGACTGAATTTGAGCTCTCCAGGTGCTTCTGAGAT   1000

1 [101 .. 106] CAACTG
2 [127 .. 132] CAAATG
3 [337 .. 342] CACCTG
4 [535 .. 540] CACCTG
5 [834 .. 839] CAGATG
6 [853 .. 858] CAGATG
7 [918 .. 923] CACATG
8 [961 .. 966] CAAGTG
9 [984 .. 989] CAGGTG
```

Figure S3: The analysis of putative E-box domains in S100A4 promoter. E-box domain is defined as sequences contain CANNTG. The bioinformatics analysis of E-box domains within S100A4 promoter was conducted by Transcriptional Regulatory Element Database (https://cb.utdallas.edu/cgi-bin/TRED/tred.cgi?process=home).
